# Cellular Imaging of Human Atherosclerotic Lesions by Intravascular Electric Impedance Spectroscopy

**DOI:** 10.1371/journal.pone.0035405

**Published:** 2012-04-11

**Authors:** Ines Streitner, Markus Goldhofer, Sungbo Cho, Ralf Kinscherf, Hagen Thielecke, Martin Borggrefe, Tim Süselbeck, Florian Streitner

**Affiliations:** 1 1st Department of Medicine-Cardiology, University Medical Centre Mannheim, Mannheim, Germany; 2 Department of Biohybride Systems, Fraunhofer Institute for Biomedical Engineering, St. Ingbert, Germany; 3 Department of Medical Cell Biology, University of Marburg, Marburg, Germany; Indiana University School of Medicine, United States of America

## Abstract

**Background:**

Newer techniques are required to identify atherosclerotic lesions that are prone to rupture. Electric impedance spectroscopy (EIS) is able to provide information about the cellular composition of biological tissue. The present study was performed to determine the influence of inflammatory processes in type Va (lipid core, thick fibrous cap) and Vc (abundant fibrous connective tissue while lipid is minimal or even absent) human atherosclerotic lesions on the electrical impedance of these lesions measured by EIS.

**Methods and Results:**

EIS was performed on 1 aortic and 3 femoral human arteries at 25 spots with visually heavy plaque burden. Severely calcified lesions were excluded from analysis. A highly flexible micro-electrode mounted onto a balloon catheter was placed on marked regions to measure impedance values at 100 kHz. After paraffin embedding, visible marked cross sections (n = 21) were processed. Assessment of lesion types was performed by Movats staining. Immunostaining for CD31 (marker of neovascularisation), CD36 (scavenger cells) and MMP-3 (matrix metalloproteinase-3) was performed. The amount of positive cells was assessed semi-quantitatively. 15 type Va lesions and 6 type Vc lesions were identified. Lesions containing abundant CD36-, CD31- and MMP-3-positive staining revealed significantly higher impedance values compared to lesions with marginal or without positive staining (CD36+455±50 Ω vs. CD36- 346±53 Ω, p = 0.001; CD31+436±43 Ω vs. CD31- 340±55 Ω, p = 0.001; MMP-3+ 400±68 Ω vs. MMP-3- 323±33 Ω, p = 0.03).

**Conclusions:**

Atherosclerotic lesions with abundant neovascularisation (CD31), many scavenger receptor class B expressing cells (CD36) or high amount of MMP-3 immunoreactivity reveal significantly higher impedance values compared to lesions with marginal or no detection of immunoreactivity. Findings suggest that inflammatory processes in vulnerable plaques affect the impedance of atherosclerotic lesions and might therefore be detected by EIS.

## Introduction

Atherosclerosis is an inflammatory disease, complicated by progressively increasing atherosclerotic plaques that eventually may rupture. Plaque rupture with subsequent thrombus formation is a major cause of serious cardiovascular events, such as unstable angina, myocardial infarction and stroke [1].

The morphologic and cellular composition of the atherosclerotic plaque is the major determinant of plaque vulnerability. Therefore, more elaborate or adjunct complementary techniques might be necessary to allow discrimination of plaque components. It has been demonstrated earlier that electric impedance spectroscopy (EIS), performed by a microelectrode-system mounted on a conventional balloon catheter, has the potential to discriminate between early stages of atherosclerosis and unaltered arteries and is able to detect neointimal proliferation after stent implantation [2], [3]. Additionally, EIS seems to be able to discriminate different plaque types in human aortic and femoral tissue [4].

The purpose of the present experimental study was to determine the influence of inflammatory plaque components on the electric impedance of potentially vulnerable atherosclerotic type Va (lipid core, thick fibrous cap) and Vc lesions (fibrous complex lesions, lipid core minimal or absent), as defined by Stary et al. [5] Impedance values were measured by a newly designed intravascular impedance balloon catheter.

## Methods

### Electrode array

**Figure 1:**
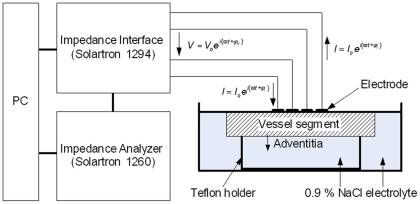
Experimental setup of the impedance measurement system.

An ultra light electrode based on polyimide was designed. For the microfabrication of the electrode structure photolithographic methods based on semiconductor technology were used. A linear array of four microelectrodes, transmission lines and terminals were integrated in an insulated polyimide of 10 µm thickness. The process technology for a polyimide-based electrode structure was described in detail by Stieglitz et al. [6] The electrode structure consisted of four platinum electrodes. The electrodes were arranged axially with a diameter of 100 µm and a spacing of 333 µm apart. The terminals of the electrodes were connected to a computer-controlled impedance measurement system. The impedance measurement system consisted of an impedance analyser SI 1260 (Solartron, Farnborough, UK) in combination with a bioimpedance interface SI 1294 (Solartron, Farnborough, UK). A sinusoidal current was fed via two outer electrodes and controlled in a way that the voltage drop across the inner electrodes was 10 mV. For all measurements the magnitude and the phase angle of the impedance were determined. Before measuring, the electrode was calibrated in saline solution (0.9%) of known electrical conductivity.

### Sample processing

1 aortic and 3 femoral human arteries were obtained during surgical procedures. The study design complies with the declaration of Helsinki and was approved by the ethics committee of the faculty of medicine (Mannheim, Germany). Written informed consent was obtained from all patients. Arteries were promptly stored in 0.9% saline solution at 4°C.

After cutting the arteries lengthwise, measuring points were marked with adventitial crosses of threads. Segments were arranged flat on a teflon holder to minimize the influence of different vessel thicknesses on the electric impedance measurements [7]. Segments were covered with 0.9% saline solution with a level just as high as the vessel wall leading to an adsorption of the electrode on the intima of the vessel wall. Thereafter, EIS was performed on 25 marked spots with heavy plaque burden. Impedance measurements were performed at 100 kHz and 37°C temperature. Obviously calcified regions were not examined. The experimental setup of the impedance measurement system is depicted in figure 1.

### Immunostaining

Each artery segment was fixed by paraformaldehyde (4%) immediately after the impedance measurements and was processed for routine paraffin embedding. Cross sections of 4 µm were cut on a kryostat and stained with haematoxylin-eosin. Out of all 25 cross sections, 4 (16%) displayed no labelling or the labelling affected the medial or the intimal vessel wall. Only correctly labelled cross sections (n = 21, 84%) were used for histological analysis. Movats pentachrom staining was performed to grade lesions according to the Stary classification of atherosclerotic lesions [5].

Histomorphological classification was conducted without knowledge of the impedance results. Type Va (lipid core, thick fibrous cap) and type Vc lesions (abundant fibrous connective tissue, while lipid is minimal or even absent) were analyzed. Immunostaining was performed using antibodies directed against CD31 (marker of neovascularisation), CD36 (scavenger receptor class B) and MMP-3 (matrix metalloproteinase-3). The amount of positive cells was assessed semi-quantitatively (no/minor vs. abundant staining).

### Statistical analysis

Results are presented as mean±standard deviation (SD). Comparisons of continuous variables were performed by using Levene statistic and 2-tailed Student's *t-*test. Categorical variables were compared using the fisher's exact test. P-values <0.05 were considered to be statistically significant. Vertical box plots were drawn of numerical data. Receiver-operating characteristic (ROC) curves were performed to estimate the test quality by assessing sensitivity and specificity values. Cut-off values were computed according to maximum sum of sensitivity and specificity estimates (Youden index). For data storage and analysis, SPSS version 11.5.1 (SPSS, Inc., Chicago, Illinois) was used.

## Results

**Figure 2:**
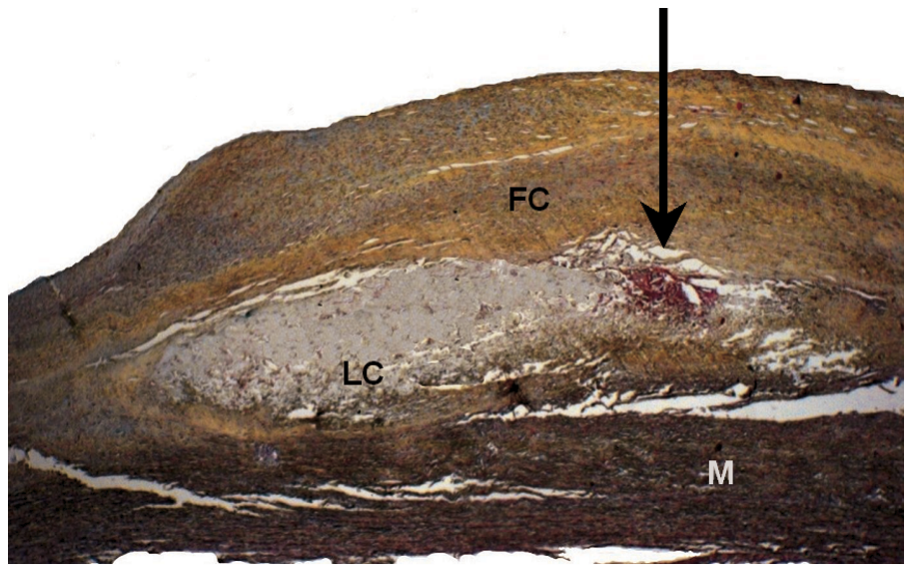
Cross section depicting an advanced lesion type Va. Movats staining, magnification 20×. LC: Characteristic lipid core. FC: Thick fibrous cap with yellow staining of collagen fibers and red-colored smooth muscle cells. M: Media. Arrow: Microhemorrhages, typically found at the lateral margin of the lipid core (red: stained fibrin).

**Figure 3:**
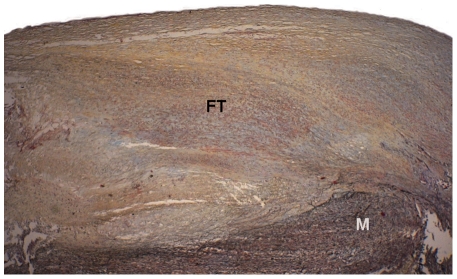
Cross section depicting an advanced lesion type Vc. Movats staining, magnification 20×. No lipid core. The lesion basically consists of fibrous connective tissue (FT) with yellow staining of collagen fibers and red-colored smooth muscle cells. M: Media.

In the present study, 15(71%) type Va lesions (Figure 2) and 6 (29%) type Vc lesions (Figure 3) were examined by EIS and by histology.

### Comparison of type Va and Vc lesions

When performing EIS, type Va lesions revealed a trend to higher mean impedance values at 100 kHz compared to Vc lesions (Va 384±69 Ω vs. Vc 325±42 Ω, p = 0.069). After immunostaining, type Va lesions displayed significantly more MMP-3 staining compared to Vc lesions (p = 0.046). Both lesion types exhibited comparable amounts of CD31- and of CD36-posititive staining (p = ns).

### Impedance and inflammation

**Figure 4:**
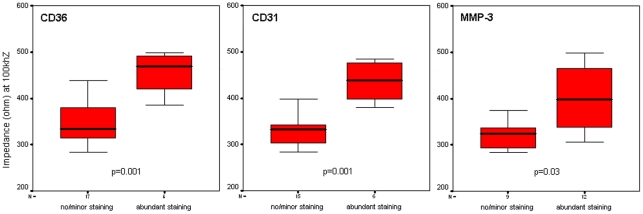
Influence of inflammation on impedance values determined by EIS (box plots).

Type Va/c lesions containing abundant CD31 as well as scavenger receptor class B expressing cells (CD36) or a high amount of MMP-3 immunoreactivity revealed significantly higher impedance values compared to lesions without or with marginal staining (CD36+(n = 4) 455±50 Ω vs. CD36-(n = 17) 346±53 Ω, p = 0.001; CD31+(n = 6) 436±43 Ω vs. CD31-(n = 15) 340±55 Ω, p = 0.001; MMP-3+(n = 12) 400±68 Ω vs. MMP-3-(n = 9) 323±33 Ω, p = 0.03). (Figure 4)

**Figure 5:**
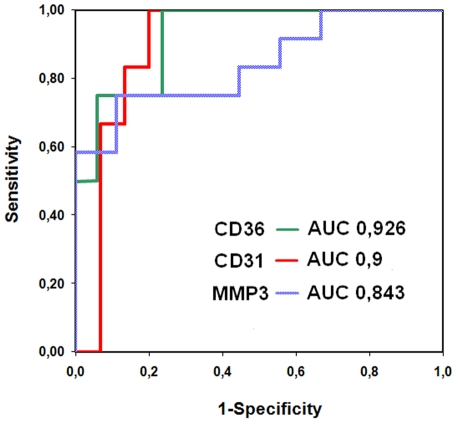
ROC curves computed by determined impedance values (at 100 kHz). ROC curves separating samples of abundant and minor/no staining with good (MMP-3) to excellent (CD31, CD36) accuracy.

Computing ROC curves, the area under the curve (AUC) was high for all markers with the highest AUC being determined for lesions with abundant CD36 staining (0.926 (p = 0.009) for CD36+lesions; 0.900 (p = 0.005) for CD31+staining; 0.843 (p = 0.009) for MMP3 staining). (Figure 5)

A cut-off value of 382 Ω displayed a sensitivity of 100% and a specificity of 76% for the detection of abundant CD36-positive lesions. Values above 382 Ω were observed in 8 (38%) of all lesions. A cut-off value of 361 Ω displayed a sensitivity of 100% and a specificity of 80% for the detection of abundant CD31-positive lesions. Values above 361 Ω were observed in 9 (42%) of all lesions. A cut-off value of 342 Ω displayed a sensitivity of 75% and a specificity of 89% for the detection of abundant MMP-3-positive lesions. Values above 342 Ω were observed in 9 (42%) of all lesions. (Table 1)

## Discussion

### Main findings

In the present study, atherosclerotic lesions type Va/Vc containing inflammatory processes identified by high amounts of MMP-3 immunoreactivity including abundant neovascularisation (CD31) and large amounts of CD36 scavenger receptor expressing cells revealed significantly higher impedance values compared to lesions either with low or without positive staining. Therefore, the results of the present study demonstrate that not just tissue structure but also qualitative tissue components like inflammatory cells and their proteolytic enzymes can influence EIS results.

**Table 1:** Value of electric impedance measurements in detecting inflammatory processes defined by different markers of inflammation.

Marker of inflammation	Cut-off value	Sensitivity	Specificity
CD31	361Ω	100%	80%
CD36	382Ω	100%	76%
MMP3	342Ω	75%	89%

### Inflammation and impedance

Morbidity and mortality from atherosclerosis is largely due to type V lesions in which disruptions of the lesion surface, hematoma or hemorrhage and thrombotic deposits have developed [5]. Factors that may play a role in causing or facilitating intimal disruptions and thrombosis, thus include the presence of inflammatory cells in lesions as well as the release of toxic substances and proteolytic enzymes by macrophages [5]. Adventitial derived vasa vasorum neovascularisation develops under the trigger of oxidized low-density lipoprotein deposits in the intima, mediated e.g. by hypoxia. Additionally, extravasation of red blood cells from leaky neovessels attracts macrophages. Macrophage erythrophagocitosis leads to cell activation at crucial sites of the plaque and macrophage-derived MMP secretion occurs [8]. MMPs are able to directly degrade extracellular matrix components and cleave many cell surface molecules and extracellular regulators. They therefore influence not only matrix remodelling but the migration, proliferation and death of vascular cells [9]. It is well known that the loss of collagen and other extracellular matrix components occurs in the highly-inflamed regions of plaque cap, thereby reducing tensile strength, as well as in the lipid core [9]. CD36 scavenger receptors, expressed on macrophages, additionally trigger cytoskeletal remodelling, important events in foam cell formation and macrophage migration [10]. These effects eventually lead to the rupture of the internal elastic lamina and fibrous cap collagenolysis, precipitating plaque rupture and thrombosis [11], [12].

The above mentioned inflammatory processes of active atherosclerotic lesions seem to have a higher impact on EIS than the tissue structure of type Va and type Vc lesions itself as lesions containing abundant CD31, CD36 or MMP-3 immunoreactivity revealed significantly higher impedance values compared to lesions without or with minor staining while the overall mean impedance values of type Va vs. type Vc lesions did not differ significantly. Therefore, EIS might be a feasible method to detect atherosclerotic lesions containing inflammatory activity.

### Experimental setup, clinical perspectives and potential limitations of EIS

The first in vitro experimental study measuring resistance of human atherosclerotic plaques was performed by Slager et al. using a spot electrode in conjunction with a large plate electrode [13]. Stiles et al. developed a computer simulation to characterize different types of atherosclerotic lesions [14]. Two years later, changes in the electric impedance conducted with four wire electrodes in bovine aorta made it possible to determine the moment when an electrode made contact with the arterial wall [15]. Subsequently, a four-point microelectrode mounted on a conventional coronary balloon catheter was newly designed, enabling intravascular measurements of small vessel areas. The performance of the balloon impedance catheter was evaluated in an atherosclerotic rabbit model. Results indicated that aortic segments with atherosclerotic plaques revealed different impedance values compared to unaltered aortic tissue [2]. Subsequently, neointimal growth after iliacal stent implantation was detected in an animal model under in-vivo conditions [3]. The next step was to examine human atherosclerotic tissue. In this setup, EIS was able to distinguish different high grade atherosclerotic lesions from another at 100 kHz frequency [4]. Although the limited number of available samples might have influenced our data, the results of the present study now demonstrate that not just plaque structure but also qualitative components like inflammatory cells and their proteolytic enzymes influence EIS results, representing a next step when utilizing this new method. However, EIS has to be performed in settings with the lowest achievable environmental influence. It is well known from earlier experiments that EIS requires a close tissue contact [7], [16]. Vessel damage might constitute an additional important limitation of the technique itself. Therefore, only spots without visible damage were analyzed in the present study and the tissue contact was kept constant. Perspectively, more has to be learned about the in vivo application of the device. The impact of blood stream and the electric properties of blood surrounding the electrodes during EIS have to be determined. The influence of the pulsatile vessel movement and of electric currents has to be assessed. Furthermore, it has to be evaluated if the method is as sensitive in the coronaries with smaller vessel diameters and lower intimal thicknesses compared to the femoral and aortic arteries under test, although simulations performed before demonstrated that inner vessel diameters did not influence measurement results [7]. However, along with further knowledge and appropriate technical development, EIS could be an additional tool to provide online in vivo information of atherosclerotic lesions in future.

### Conclusion

Atherosclerotic lesions with abundant neovascularisation (CD31), many scavenger receptor class B expressing cells (CD36) or high amount of MMP-3 immunoreactivity reveal significantly higher impedance values compared to lesions with marginal or no detection of immunoreactivity. Our findings suggest that inflammatory processes in vulnerable plaques affect the impedance of atherosclerotic lesions and might therefore be detected by EIS.
